# Monitoring the formation of cholesterol oxidation products in model systems using response surface methodology

**DOI:** 10.1186/s12944-015-0074-6

**Published:** 2015-07-23

**Authors:** Joong-Seok Min, Sang-Ok Lee, Muhammad Issa Khan, Dong Gyun Yim, Kuk-Hwan Seol, Mooha Lee, Cheorun Jo

**Affiliations:** CJ Food Research Center, Seoul, 152-050 Korea; Department of Agricultural Biotechnology, Center for Food and Bioconvergence, and Research Institute of Agriculture and Life Science, Seoul National University, Seoul, 51-921 Korea; National Institute of Food Science and Technology, University of Agriculture, Faisalabad, 38040 Pakistan; Department of Health Administration and Food Hygiene, Jinju Health College, Jinju, 660-757 Korea; National Institute of Animal Science, RDA, Cheonan, 331-801 Korea

**Keywords:** Cooking conditions, pH, Unsaturated fatty acids, COPs

## Abstract

**Background:**

Cholesterol oxidation products (COPs) are produced during thermal processing of animal origin foods and are considered to have negative health impacts. The model systems are helpful to understand the impact of various factors on oxidation changes in foods during cooking process.

**Methods:**

The study presented herein investigates the effects of pH, presence of unsaturated fatty acids, and heat on the formation of cholesterol oxidation products (COPs). Two model systems were designed to investigate the formation of cholesterol oxidation products in different lipid environments. The cholesterol oxides produced were quantified using gas chromatography.

**Results:**

The level of cholesterol oxidation products decreased significantly at higher pH (above 5.8) and shorter heating time (3 h). The presence of unsaturated fatty acids (linoleic and oleic acids) significantly increased the amount of COPs under low-temperature heating conditions (100 °C and 1 h) but did not affect the production of COPs at higher temperature (150 °C). Increasing the temperature to 200 °C significantly increased the amount of COPs during the first hour of heating and this amount decreased upon further heating. The most frequently observed COPs in samples were α-epoxide, 20α-hydroxycholesterol, and 25-hydroxycholesterol.

**Conclusions:**

In conclusion, pH below 5.8, presence of unsaturated fatty acid, and high cooking temperature (>150 °C) leads to increased production of cholesterol oxidation products.

## Introduction

Cholesterol is a biologically important compound that is widely present in animal-derived foods. Storing cholesterol in the presence of oxidants (light, oxygen, and catalysts) leads to the formation of cholesterol oxidation products (COPs). COPs are considered to be more detrimental to arterial cells than is pure cholesterol and are directly linked to atherosclerosis and coronary heart disease [1]. Additionally, COPs inhibit the biosynthesis [2] and dietary uptake [3] of cholesterol. Therefore, COPs are considered to be cytotoxic, mutagenic, and carcinogenic [4]. The main causes of cholesterol oxidation in animal-derived foods are cooking, dehydration, and deep frying [5].

Healthy human plasma contains 12.6 mg/L of COPs [6] and ingestion of foods containing COPs increases these levels in plasma and leads to deleterious health effects. More than 80 COPs have been identified, and those most commonly found in food are 7-ketocholesterol (7-keto), 6-ketocholesterol (6-keto), 7α-hydroxy-cholesterol (7α-OH), 7β-hydroxycholesterol (7β-OH), 5,6α-epoxycholesterol (5,6α-EP), 5,6β-epoxycholesterol (5,6β-EP), 25-hydroxycholesterol (25-OH), 20-hydroxycholesterol (20-OH), and cholestanetriol (triol). COPs are formed when animal-derived foods are subjected to the following processes: heating and cooking [7], dehydration [8], storage [9], and irradiation [10].

The major factors that influence the formation of COPs are heat, pH, light, oxygen, water activity, and the presence of unsaturated fatty acids (UFAs). The UFAs in meat are very susceptible to peroxidation, even under mild ambient conditions, and consequently yield both free and peroxy radicals [11]. Therefore, it is reasonable to suspect that oxidation of cholesterol is accelerated in the presence of peroxydized lipids [12]. Saldanha et al. [13] reported a decreased level of polyunsaturated fatty acids (PUFAs) with a decrease in the total amount of cholesterol during the grilling of sardines. The synergistic decrease in the levels of cholesterol and PUFAs provides evidence that cholesterol oxidation is promoted by the oxidation of PUFAs. However, the environmental conditions to promote the production of COPs still need to be determined.

Accordingly, the present study was designed to investigate the effects of pH and unsaturated fatty acids on the formation of COPs under different heating conditions in model systems.

## Materials and methods

### Reagents and solutions

Cholesterol, linoleic acid, oleic acid, cholesterol oxide standards (7-keto, 6-keto, 7α-OH, 7β-OH, 5,6α-EP, 5,6β-EP, 25-OH, 20-OH, and triol), butylated hydroxytoluene (BHT), pyridine, and silicic acid (100 mesh) were purchased from Sigma-Aldrich Co., LLC (Seoul, Korea). *Bis*-[trimethylsilyl]-trifluoroacetamide (BSTFA) + 1 % trimethylchlorosilane (TMCS) was obtained from Supelco (Bellefonte, PA, USA). HPLC grade hexane, ethyl acetate, acetone, methanol, and chloroform, Celite 545, and calcium phosphate (CaHPO_4_.2H_2_O) were purchased from Fisher Scientific Co. (Malvern, PA, USA).

### Model system-I

Model system-I was designed with suitable emulsions to assess the influence of pH on cholesterol oxidation using the method of Miyako et al. [14] which is described in this section. Borate buffer (0.1 M, 10 mL) and cholesterol (50 mg) were mixed in a vortex mixer (G560, Scientific Industries Inc., NY, USA) for 20 s and then placed in a sonicator (Braun GmbH, Kronberg, Germany) for 10 min. The pH of the resulting emulsions was adjusted to 5.8, 6.7, and 7.6 using either HCl or NaOH. The emulsions (10 mL) were stored at 4 °C for 10 d and then filtered through a Whatman No. 1 (Whatman Inc., Clifton, NJ). BHT (7.2 %, 50 μL) was added to the filtered emulsions to prevent further oxidation during analysis of the COPs. The amounts of COPs were measured using gas chromatography (GC) according to the method described by Lee et al. [10].

### Model system-II

Model system-II was designed to assess the influence of unsaturated fatty acids on cholesterol oxidation. Cholesterol (50 mg) was dissolved in chloroform (1 mL) and placed in a 10 mL test tube. The solvent was evaporated under an atmosphere of nitrogen to obtain a thin cholesterol film. Afterwards, cholesterol was mixed with unsaturated fatty acids (linoleic acid or oleic acid) in ratios of 1:20 and 1:100, respectively. The mixtures were heated for 1, 3, 5, and 10 h at various temperatures (100, 150, and 200 °C) in an oil bath. After the heat treatments, BHT (7.2 %, 50 μL) was added to the filtered emulsions to inhibit further oxidation during analysis of the COPs. The amounts of COPs were measured using gas chromatography (GC) according to the method described by Lee et al. [10].

### Response surface methodology

The effects of the heating conditions (heating temperature and heating time) on the production of COPs were monitored using the response surface methodology experiment designed by Gontard et al. [15]. The experimental design consisted of two factors with varying levels that were replicated thrice at the center point [16]. The independent variables, the coded variables, and their levels of interest for this research are presented in Table 1.

**Table 1:** Conditions with different combinations of independent variable used in central composite rotatable second order design for response surface methodology

Treatment No.	X1	*X*2	Heating Temperature (t)	Heating Time (T)
1	−1	−1	100	1
2	−1	−0.5	100	3
3	−1	−0.1	100	5
4	−1	1	100	10
5	0	−1	150	1
6	0	−0.5	150	3
7	0	−0.1	150	5
8	0	1	150	10
9	1	−1	200	1
10	1	−0.5	200	3
11	1	−0.1	200	5
12	1	1	200	10

### Statistical analysis

Data were analyzed with Statistical Analysis System (SAS) software to evaluate the effects of pH, unsaturated fatty acids, and heating conditions on the formation of COPs. All the experimental procedures were triplicated. The amount of COPs (Y) was subjected to analyze for dependent variables (responses). Means values of triplicate determinations were analyzed to fit the following second-order polynomial models to Y variables [15].

$$ \mathrm{Y} = {\mathrm{b}}_0 + {\mathrm{b}}_1{\mathrm{X}}_1 + {\mathrm{b}}_2{\mathrm{X}}_2 + {\mathrm{b}}_{12}{\mathrm{X}}_1{\mathrm{X}}_2 + {\mathrm{b}}_{11}{{\mathrm{X}}_1}^2 + {\mathrm{b}}_{22}{{\mathrm{X}}_2}^2 $$

Where X_1_ and *X*_2_ correspond to independent variables (heating time and temperature) and b_n_ represent corresponding regression coefficient. Response surface and contour plots were developed using fitted polynomial equations.

## Results and discussion

### Effects of pH on the formation of COPs

Cholesterol oxidation products (COPs), or oxysterols, have received increasing attention for the following biological roles: diagnostic biomarker of oxidative stress, intermediates in bile acid biosynthesis, messengers for cell signaling, and cholesterol transport [17]. Cholesterol is more susceptible to autoxidation in the liquid state [18] and is oxidized by both enzymatic and non-enzymatic mechanisms [17]. The results illustrating the impact of pH on the formation of COPs are depicted in Table 2. Three main cholesterol oxidation products were detected in samples stored for 10 d at 4 °C: 20α-OH, 25-OH, and α-epoxide. Triol was additionally found in control samples. In the current model system, cholesterol was autoxidized by a non-enzymatic mechanism. Results from statistical analysis studies showed that there were no significant differences observed between the pH treatments (5.8 to 7.6), but a significant difference was observed between the control sample and the pH-treated samples. The control sample (cholesterol without borate buffer) exhibited higher levels of 20α-OH, 25-OH, and α-epoxide than samples with borate buffer. It is evident from these results that a pH range between 5.8 and 7.6 does not affect the oxidation of cholesterol at refrigeration temperatures. Meanwhile, lower pH levels (5.6 and 5.8) of the oxidation medium promote increased lipid oxidation [19]; accelerated lipid oxidation was observed in carcasses exposed to pH levels below 5.8 [20]. The pronounced discoloration of carcasses during chilled storage below pH 5.8 may result from higher rates of oxymyoglobin oxidation to produce metmyoglobin [21]. Cholesterol oxidation in meat and meat products has been observed during frozen storage [22] as well as during heating [18]. Kowale et al. [23] observed faster changes in cholesterol oxides for refrigerated mutton compared to frozen mutton; this observation was attributed to the presence of free radicals, which resulted from the oxidation of UFAs.

**Table 2:** The contents of COPs under different pH condition after storage at 4 °C for 10 days

Treatment COPs (mg)	Control	pH 5.8	pH 6.7	pH 7.6
7β-OH	Nd	nd	nd	nd
20α-OH^*^	0.51 ± 0.07^a^	0.43 ± 0.02^ab^	0.37 ± 0.01^b^	0.38 ± 0.06^b^
25-OH^***^	0.13 ± 0.01^a^	0.07 ± 0.01^b^	0.06 ± 0.00^b^	0.06 ± 0.02^b^
Triol^***^	0.02 ± 0.00^a^	n.d.^b^	n.d.^b^	n.d.^b^
α-epoxide	0.04 ± 0.01	0.03 ± 0.01	0.03 ± 0.01	0.03 ± 0.00
7-keto	Nd	nd	nd	nd
Total amount of COPs/cholesterol (%) ^**^	1.40 ± 0.17^a^	1.06 ± 0.08^b^	0.91 ± 0.05^b^	0.95 ± 0.16^b^

*7β-OH* 7β-hydroxycholesterol, *20α-OH* 7α-hydroxy-cholesterol, *25-OH* 25-hydroxycholesterol, *Triol* Cholestanetriol, *α-epoxide* 5,6α-epoxycholesterol, *7-keto* 7-ketocholesterol

^*^: *P* < 0.05, ^**^: *P* < 0.01, ^***^: *P* < 0.001

^a, b^ Means ± SE with different superscript in the same row differ significantly

### Effects of linoleic and oleic acids on the formation of COPs

The focus of this study was to assess the impact of heating conditions and the addition of unsaturated fatty acids on COP production in model systems. Samples were heated at 100, 150, and 200 °C for 1, 3, 5, 7 and 10 h; some samples did not contain added unsaturated fatty acids while others contained cholesterol with either linoleic or oleic acid in ratios of 1:20 and 1:100, respectively. The results regarding the changes in the COP contents (mg) of samples with or without linoleic or oleic acid heated at 100 °C for 1 to 10 h are shown in Table 3. The COPs 25-OH, α-epoxide, 7-keto were produced when cholesterol alone was heated at 100 °C for 1 h. 7β-OH was detected after 3 and 5 h of heating, while 20α-OH was detected after 3 and 10 h of heating. However, the COPs α-epoxide and 7-keto were not detected after 5 h of heating. The amounts of 7β-OH were significantly higher in samples CL_1_, CL_2_, CO_1_ and CO_2_ after heating for 1 h compared to other heating times (3, 5, 7, and 10 h), while small amounts of 7β-OH were detected in control samples only after heating for 3 and 5 h (Table 3). Chien et al. [24] studied the kinetics of cholesterol oxidation during heating by selecting five major COPs namely 7-OOH (7α- and 7β-hydroperoxycholesterol), 7-ketocholesterol, 7-OH (7α- and 7β-hydroxycholesterol), 5, 6–epoxy (5, 6 α- and 5, 6 β-epoxycholesterol), and triol (5α-cholestane-3β, 5,6β-triol). They observed that concentration of COPs increased with the increase in heating time.

**Table 3:** The changes in COPs contents (mg) in cholesterol with or without unsaturated fatty acids during heating at 100 °C

Treat	Hour	7β-OH^***^	20α-OH^***^	25-OH^***^	Triol^***^	α-epoxide^***^	7-keto^***^	Total amount of COPs
C	1	n.d.^d^	n.d.^f^	0.07 ± 0.00^bc^	n.d.^d^	0.02 ± 0.01^ghi^	0.03 ± 0.00^bc^	0.11 ± 0.002^ef^
	3	0.08 ± 0.01^d^	0.11 ± 0.01^e^	0.05 ± 0.01^bcd^	n.d.^d^	0.02 ± 0.00^ghi^	0.05 ± 0.00^b^	0.31 ± 0.03^ef^
	5	0.01 ± 0.00^d^	n.d.^f^	0.01 ± 0.00^f^	n.d.^d^	n.d.^i^	n.d.^c^	0.01 ± 0.001^f^
	10	n.d.^d^	0.02 ± 0.00^f^	0.01 ± 0.00^ef^	n.d.^d^	n.d.^i^	n.d.^c^	0.03 ± 0.01^f^
CL_1_	1	1.47 ± 0.18^b^	0.21 ± 0.03^c^	0.06 ± 0.01^bc^	n.d.^d^	0.02 ± 0.00^fgh^	n.d.^c^	1.76 ± 0.22^b^
(1:20)	3	n.d.^d^	0.02 ± 0.00^f^	0.003 ± 0.00^f^	n.d.^d^	0.13 ± 0.01^b^	n.d.^c^	0.15 ± 0.01^ef^
	5	n.d.^d^	0.03 ± 0.01^f^	0.01 ± 0.00^f^	n.d.^d^	0.16 ± 0.02^a^	n.d.^c^	0.19 ± 0.03^ef^
	10	n.d.^d^	0.26 ± 0.08^b^	0.06 ± 0.01^bc^	n.d.^d^	0.03 ± 0.01^efgh^	0.35 ± 0.09^a^	0.69 ± 0.18^d^
CL_2_	1	1.29 ± 0.08^b^	0.17 ± 0.01^d^	0.08 ± 0.01^b^	0.02 ± 0.00^a^	0.03 ± 0.00^defg^	0.06 ± 0.00^b^	1.64 ± 0.10^b^
(1:100)	3	n.d.^d^	0.002 ± 0.00^f^	0.16 ± 0.07^a^	0.01 ± 0.00^b^	0.05 ± 0.01^cd^	n.d.^c^	0.22 ± 0.09^ef^
	5	0.01 ± 0.00^d^	0.01 ± 0.00^f^	0.004 ± 0.00^f^	0.01 ± 0.00^c^	n.d.^i^	n.d.^c^	0.04 ± 0.004^f^
	10	n.d.^d^	0.01 ± 0.00^f^	0.002 ± 0.00^f^	n.d.^d^	n.d.^i^	n.d.^c^	0.01 ± 0.002^f^
CO_1_	1	0.66 ± 0.03^c^	0.29 ± 002^b^	0.04 ± 0.00^cde^	n.d.^d^	0.01 ± 0.00^hi^	n.d.^c^	1.01 ± 0.05^c^
(1:20)	3	n.d.^d^	0.0003 ± 0.00^f^	0.01 ± 0.00^f^	n.d.^d^	0.04 ± 0.00^def^	n.d.^c^	0.05 ± 0.003^f^
	5	n.d.^d^	0.02 ± 0.00^f^	0.01 ± 0.00^f^	n.d.^d^	0.12 ± 0.02^b^	n.d.^c^	0.15 ± 0.03^ef^
	10	n.d.^d^	0.36 ± 0.05^a^	0.02 ± 0.00^def^	n.d.^d^	0.03 ± 0.00^defg^	n.d.^c^	0.42 ± 0.06^de^
CO_2_	1	3.79 ± 0.63^a^	0.18 ± 0.02^cd^	0.06 ± 0.01^bc^	0.02 ± 0.00^a^	0.03 ± 0.00^def^	0.05 ± 0.00^b^	4.12 ± 0.67^a^
(1:100)	3	n.d.^d^	0.004 ± 0.00^f^	0.006 ± 0.00^f^	n.d.^d^	0.06 ± 0.03^c^	n.d.^c^	0.07 ± 0.03^f^
	5	n.d.^d^	0.01 ± 0.00^f^	0.003 ± 0.00^f^	n.d.^d^	0.04 ± 0.00^de^	n.d.^c^	0.05 ± 0.01^f^
	10	n.d.^d^	0.01 ± 0.00^f^	n.d.^f^	n.d.^d^	n.d.^i^	n.d.^c^	0.01 ± 0.001^f^

*C* cholesterol (50 mg), *L* linoleic acid, O: oleic acid, *CL*_*1*_ cholesterol (50 mg) : linoleic acid (1 g) = 1 : 20, *CL*_*2*_ cholesterol (50 mg) : linoleic acid (5 g) =1 : 100, *CO*_*1*_ cholesterol (50 mg) : oleic acid (1 g) = 1 : 20, *CO*_*2*_ cholesterol (50 mg) : oleic acid (5 g) = 1 : 100

*7β-OH* 7β-hydroxycholesterol, *20α-OH* 7α-hydroxy-cholesterol, *25-OH* 25-hydroxycholesterol, *Triol* Cholestanetriol, *α-epoxide* 5,6α-epoxycholesterol, *7-keto*: 7-ketocholesterol

^***^ *P* < 0.001. *n.d* not detected

^a–i^ Means ± SE with different superscript in the same column differ significantly

The addition of unsaturated fatty acids significantly affected the production of 20α-OH and the greatest amount (0.36 mg) was observed in sample CO_1_ after 10 h of heating. In the control sample (cholesterol only), the greatest amount of COP 25-OH was detected after 1 h of heating; the tendency of the control sample to produce 25-OH was similar to that of CL_1_, CO_1_ and CO_2_. Meanwhile, the greatest amount of 25-OH in CL_2_ was produced after 3 h of heating. The greatest amounts of triol were detected in CL_2_ and CO_2_ after heating for 1 h, yet triol was not detected in C, CL_1_ and CO_1_. The concentration of α-epoxide varied significantly from ND to 0.16 mg. The COP α-epoxide was not detected in C (5 and 10 h of heating), CL_2_ (5 and 10 h of heating), or CO_2_ (10 h of heating). Meanwhile, the greatest amount of α-epoxide (0.16 mg) was observed in CL_1_ after 5 h heating. The COP 7-keto was found in C, CL_2_ and CO_2_ after heating for 1 h, but it was not observed in CL_1_ and CO_1_. Heating for longer time periods did not result in additional production of keto in either C (after 5 h) or CL_2_ and CO_2_ (after 3 h), yet a higher concentration of keto (0.35 mg) was detected in CL_1_ after 10 h of heating. It has been reported that heating cholesterol in the presence of oxygen proceeds to oxidation and degradation [25]. The two major routes of oxidation are C-7 oxidation and epoxidation. The oxidation reaction is initiated by the attack of free radicals on cholesterol, which produces 7-OOH according to a second-order pathway; the 7-OOH formation rate equation was described in a report by Chien et al. [25]. In the current study, the presence of UFAs (linoleic and oleic acid) in the reaction medium presumably allows the radical-mediated peroxidation reactions to proceed [17].

The results regarding the production of COPs in cholesterol samples heated at 150 °C for 1 to 10 h with or without UFAs (linoleic and oleic acid) are presented in Table 4. The cholesterol samples without UFAs (control) produced the COPs 20α-OH, 25-OH, α-epoxide, and 7-keto after heating for 1 h. Triol was not detected when samples were heated longer than 1 h, while 20α-OH, 25-OH, α-epoxide, and 7-keto were produced when samples were subjected to longer heating times (up to 10 h). The COPs 20α-OH, 25-OH, α-epoxide, and 7-keto were also found in samples with UFAs (CL_1_, CL_2_, CO_1_ and CO_2_) after heating for 1 h, as well as 10 h. In most samples, the highest COP production was observed after heating for 1 h. Chien et al. [25] observed a sharp decrease in cholesterol between 1.5 to 6 min of heating, which resulted in increased COP production, while prolonged heating resulted in a gradual decrease in cholesterol content. The residual percentage of cholesterol was 33.3 % after 30 min of heating [25] and greater losses were observed after 60 min (50.2 %) and 90 min (63.8 %) of heating [18]. These results suggest that the chances of cholesterol oxidation remain at 33.3 % of the initial level after 30 min of heating. Similarly, Saldanha et al. [13] reported a decrease in cholesterol content from 342 to 282 mg/100 g when a fresh sardine was grilled at 165 °C.

**Table 4:** The changes in COPs contents (mg) in cholesterol with or without unsaturated fatty acids during heating at 150 °C

Treat	Hour	7β-OH^***^	20α-OH^***^	25-OH^***^	Triol^***^	α-epoxide^***^	7-keto^***^	Total amount of COPs
C	1	n.d.^d^	0.25 ± 0.01^b^	0.06 ± 0.00^ef^	0.55 ± 0.05^a^	0.46 ± 0.01^a^	1.29 ± 0.16^c^	2.60 ± 0.22^a^
	3	n.d.^d^	n.d.^l^	0.10 ± 0.01^bc^	n.d.^b^	0.46 ± 0.02^a^	1.80 ± 0.01^a^	2.36 ± 0.04^a^
	5	n.d.^d^	0.16 ± 0.02^de^	0.10 ± 0.00^b^	n.d.^b^	0.25 ± 0.06^b^	1.19 ± 0.33^cd^	1.71 ± 0.42^bc^
	10	n.d.^d^	0.09 ± 0.06^gh^	0.10 ± 0.04^b^	n.d.^b^	0.25 ± 0.08^b^	1.48 ± 0.11^b^	1.92 ± 0.30^b^
CL_1_	1	n.d.^d^	0.27 ± 0.01^b^	0.10 ± 0.02^bc^	n.d.^b^	0.04 ± 0.01^efgh^	0.19 ± 0.02^ef^	0.60 ± 0.05^d^
(1:20)	3	n.d.^d^	0.11 ± 0.03^fg^	0.03 ± 0.01^ghi^	n.d.^b^	0.10 ± 0.02^cd^	0.15 ± 0.04^f^	0.39 ± 0.09^de^
	5	n.d.^d^	0.07 ± 0.02^hi^	0.02 ± 0.00^ghij^	n.d.^b^	0.05 ± 0.01^efg^	0.10 ± 0.01^f^	0.24 ± 0.03^de^
	10	2.34 ± 0.60^a^	0.02 ± 0.00^jkl^	0.004 ± 0.00^J^	n.d.^b^	0.06 ± 0.02^def^	n.d.^f^	2.45 ± 0.62^a^
CL_2_	1	n.d.^d^	0.17 ± 0.01^cd^	0.08 ± 0.01^bcd^	n.d.^b^	0.02 ± 0.00^fgh^	0.12 ± 0.04^f^	0.40 ± 0.05^de^
(1:100)	3	n.d.^d^	0.11 ± 0.01^fg^	0.08 ± 0.01^cde^	n.d.^b^	0.03 ± 0.00^fgh^	0.13 ± 0.01^f^	0.35 ± 0.03^de^
	5	n.d.^D^	0.05 ± 0.01^ij^	0.02 ± 0.00^hij^	n.d.^b^	0.02 ± 0.01^fgh^	0.04 ± 0.01^f^	0.13 ± 0.03^de^
	10	1.40 ± 0.21^b^	0.01 ± 0.00^kl^	0.01 ± 0.00^ij^	n.d.^b^	0.04 ± 0.01^fgh^	0.04 ± 0.02^f^	1.50 ± 0.25^bc^
CO_1_	1	n.d.^d^	0.37 ± 0.02^a^	0.06 ± 0.01^ef^	n.d.^b^	0.02 ± 0.01^fgh^	0.08 ± 0.01^f^	0.53 ± 0.04^de^
(1:20)	3	n.d.^d^	0.13 ± 0.01^ef^	0.04 ± 0.01^fg^	n.d.^b^	0.11 ± 0.01^c^	n.d.^f^	0.29 ± 0.04^de^
	5	n.d.^d^	0.04 ± 0.00^ijk^	0.03 ± 0.01^ghi^	n.d.^b^	0.09 ± 0.02^cde^	n.d.^f^	0.16 ± 0.03^de^
	10	1.49 ± 0.38^b^	0.0003 ± 0.00^l^	0.009 ± 0.00^ij^	n.d.^b^	0.04 ± 0.01^fgh^	1.06 ± 0.17^d^	2.60 ± 0.56^a^
CO_2_	1	n.d.^d^	0.20 ± 0.02^c^	0.07 ± 0.02^de^	n.d.^b^	0.03 ± 0.01^fgh^	0.07 ± 0.00^f^	0.36 ± 0.04^de^
(1:100)	3	n.d.^d^	0.10 ± 0.01^fg^	0.03 ± 0.01^fgh^	n.d.^b^	0.01 ± 0.01^gh^	0.05 ± 0.02^f^	0.20 ± 0.05^de^
	5	n.d.^d^	0.04 ± 0.00^ijkl^	0.02 ± 0.00^ghij^	n.d.^b^	n.d.^h^	n.d.^f^	0.06 ± 0.01^e^
	10	0.84 ± 0.30^c^	0.01 ± 0.00^kl^	0.17 ± 0.01^a^	n.d.^b^	0.02 ± 0.00^fgh^	0.35 ± 0.11^e^	1.38 ± 0.42^c^

*C* cholesterol (50 mg), *L* linoleic acid, O: oleic acid, *CL*_*1*_ cholesterol (50 mg) : linoleic acid (1 g) = 1 : 20, *CL*_*2*_ cholesterol (50 mg) : linoleic acid (5 g) =1 : 100, *CO*_*1*_ cholesterol (50 mg) : oleic acid (1 g) = 1 : 20, *CO*_*2*_ cholesterol (50 mg) : oleic acid (5 g) = 1 : 100

*7β-OH* 7β-hydroxycholesterol, *20α-OH* 7α-hydroxy-cholesterol, *25-OH* 25-hydroxycholesterol, *Triol* Cholestanetriol, *α-epoxide* 5,6α-epoxycholesterol, *7-keto* 7-ketocholesterol

^***^ *P* < 0.001. *n.d* not detected

^a–l^ Means ± SE with different superscript in the same column differ significantly

The presence of either linoleic or oleic acid in the reaction medium produced interesting data, which are presented in Table 4. The presence of UFAs (linoleic and oleic acid) induced cholesterol to produce 7β-OH after 10 h of heating at 150 °C, and this did not proceed in the absence of UFAs. Further, the content of 7β-OH was higher in samples with added linoleic acid, CL_1_ (2.34 mg) and CL_2_ (1.40 mg), than in samples with added oleic acid, CO_1_ (1.49 mg) and CO_2_ (0.84 mg). These results are attributed to the different number of double bonds in linoleic acid (18:2) and oleic acid (18:1), which accelerate the oxidation of cholesterol. Higher levels of 20α-OH were observed in samples containing all combinations of UFAs and cholesterol when heated for 1 h at 150 °C. The total amounts of COPs after heating at 150 °C were higher in the control samples than in those with unsaturated fatty acids. This trend of COP production at 150 °C was different from samples heated at 100 °C, in which the addition of UFAs increased the total amounts of COPs.

The data shown in Table 5 describe the change in COP production in samples with or without UFAs (linoleic and oleic acid) heated at 200 °C for 1 to 10 h. The COP 7β-OH was not detected in control samples (cholesterol) after heating at 200 °C for 1 h, while samples with added UFAs (linoleic and oleic acid) exhibited the highest levels of 7β-OH after heating at 200 °C for 1 h. Longer heating times (3, 5, 7 and 10 h) resulted in decreased production of 7β-OH. The COP triol was detected in samples containing linoleic acid (CL_1_ and CL_2_) but not in the control sample or in the samples containing oleic acid. The control sample (C) had a significantly higher content of 7-keto than other samples subjected to various heating times (P < 0.001), and 7-keto was the sole COP observed when the sample was heated at 200 °C. These results agree with those reported by Osada et al. [26], which revealed that 7-keto was the most predominant oxidized cholesterol produced when cholesterol was heated in the presence of different fats. The levels of 20α-OH were higher in samples heated for l h than in those heated for longer times (3, 5 and 10 h) at 200 °C. The levels of 25-OH, α-epoxide, and 7-keto in samples containing linoleic acid (CL_1_ and CL_2_) gradually increased as the heating time increased, and a similar result was observed for 7-keto in the CO_2_ samples. Bascoul et al. [27] reported that the presence of unsaturated fatty acids in tallow accelerated the formation of oxidized cholesterol compared to when cholesterol alone was heated [28]. These results reflect that unsaturated fatty acids are not resistant to oxidation.

**Table 5:** The changes in COPs contents (mg) in cholesterol with or without unsaturated fatty acids during heating at 200 °C

Treat	Hour	7β-OH^***^	20α-OH^***^	25-OH^***^	Triol^***^	α-epoxide^***^	7-keto^***^	Total amount of COPs
C	1	n.d.^e^	0.18 ± 0.01^a^	0.10 ± 0.02^bc^	n.d.^c^	0.17 ± 0.01^a^	0.88 ± 0.12^bc^	1.33 ± 0.16^def^
	3	n.d.^e^	0.18 ± 0.01^a^	0.09 ± 0.01^cd^	n.d.^c^	0.11 ± 0.02^bc^	0.94 ± 0.17^b^	1.32 ± 0.21^def^
	5	n.d.^e^	0.08 ± 0.03^b^	0.08 ± 0.01^e^	n.d.^c^	0.12 ± 0.03^b^	1.10 ± 0.11^a^	1.38 ± 0.17^def^
	10	n.d.^e^	0.03 ± 0.01^e^	0.06 ± 0.01^e^	n.d.^c^	0.09 ± 0.03^bcd^	0.82 ± 0.08^bc^	1.00 ± 0.12^efg^
CL_1_	1	5.75 ± 0.40^b^	0.05 ± 0.01^c^	0.02 ± 0.00^fg^	n.d.^c^	0.08 ± 0.04^cdef^	0.09 ± 0.03^ef^	5.99 ± 0.48^b^
(1:20)	3	0.55 ± 0.02^de^	0.002 ± 0.00^F^	0.08 ± 0.02^de^	n.d.^c^	0.10 ± 0.06^bc^	0.10 ± 0.02^ef^	0.83 ± 0.12^efgh^
	5	0.24 ± 0.02^de^	n.d.^f^	0.10 ± 0.01^bc^	n.d.^c^	0.18 ± 0.02^a^	0.20 ± 0.09^e^	0.72 ± 0.14^fgh^
	10	0.18 ± 0.04^de^	n.d.^f^	0.11 ± 0.01^ab^	0.02 ± 0.01^b^	0.12 ± 0.02^b^	0.11 ± 0.01^ef^	0.54 ± 0.08^gh^
CL_2_	1	3.33 ± 0.67^c^	0.04 ± 0.00D^e^	0.02 ± 0.00^fg^	n.d.^c^	0.02 ± 0.01^gh^	n.d.^f^	3.41 ± 0.68^c^
(1:100)	3	0.19 ± 0.02^de^	0.002 ± 0.00^f^	0.01 ± 0.00^gh^	0.02 ± 0.01^b^	0.03 ± 00.01^gh^	0.03 ± 0.01^f^	0.28 ± 0.04^h^
	5	0.40 ± 0.08^de^	0.002 ± 0.00^f^	0.03 ± 0.00^fg^	0.03 ± 0.00^a^	0.06 ± 0.00^defg^	0.04 ± 0.00^f^	0.56 ± 0.09^gh^
	10	0.29 ± 0.02^de^	n.d.^f^	0.03 ± 0.01^f^	0.03 ± 0.00^a^	0.08 ± 0.01^cde^	n.d.^f^	0.44 ± 0.04^gh^
CO_1_	1	6.51 ± 0.97^a^	0.05 ± 0.01^cd^	0.06 ± 0.01^e^	n.d.^c^	0.04 ± 0.01^gh^	0.77 ± 0.08^c^	7.43 ± 1.08^a^
(1:20)	3	0.65 ± 0.02^d^	n.d.^f^	0.13 ± 0.01^a^	n.d.^c^	0.04 ± 0.01^fgh^	0.90 ± 0.13^bc^	1.71 ± 0.17^d^
	5	0.49 ± 0.09^de^	0.001 ± 0.00^f^	0.04 ± 0.01^f^	n.d.^c^	0.02 ± 0.00^gh^	n.d.^f^	0.54 ± 0.11^gh^
	10	0.21 ± 0.03^de^	n.d.^f^	n.d.^h^	n.d.^c^	0.01 ± 0.00^h^	n.d.^f^	0.22 ± 0.03^h^
CO_2_	1	3.08 ± 0.33^c^	0.04 ± 0.01^cde^	0.03 ± 0.01^f^	n.d.^c^	0.02 ± 0.01^gh^	n.d.^f^	3.18 ± 0.35^c^
(1:100)	3	0.24 ± 0.02^de^	n.d.^f^	0.06 ± 0.01^e^	n.d.^c^	0.02 ± 0.01^gh^	0.33 ± 0.06^d^	0.65 ± 0.10^gh^
	5	0.13 ± 0.03^de^	n.d.^f^	n.d.^h^	n.d.^c^	0.05 ± 0.01^efgh^	0.37 ± 0.02^d^	0.55 ± 0.06^gh^
	10	0.14 ± 0.03^de^	n.d.^f^	n.d.^h^	n.d.^c^	0.03 ± 0.01^gh^	0.42 ± 0.10^d^	0.58 ± 0.13^gh^

*C* cholesterol (50 mg), *L* linoleic acid, *O* oleic acid, *CL*_*1*_ cholesterol (50 mg): linoleic acid (1 g) = 1 : 20, *CL*_*2*_ cholesterol (50 mg) : linoleic acid (5 g) =1 : 100, CO_1_: cholesterol (50 mg) : oleic acid (1 g) = 1 : 20, *CO*_*2*_ cholesterol (50 mg): oleic acid (5 g) = 1 : 100

*7β-OH* 7β-hydroxycholesterol, *20α-OH* 7α-hydroxy-cholesterol, *25-OH* 25-hydroxycholesterol, *Triol* Cholestanetriol, *α-epoxide* 56α-epoxycholesterol, *7-keto* 7-ketocholesterol

^***^ *P* < 0.001, *n.d* not detected

^a–h^ Means ± SE with different superscript in the same column differ significantly

The data presented in Tables 3, 4, and 5 depict the total amounts of COPs generated in cholesterol samples with or without unsaturated fatty acids heated at 100, 150, and 200 °C for 1 to 10 h. The production rate of COPs was 0.01 − 8.25 %, higher in cholesterol samples with added unsaturated fatty acids than that in cholesterol samples without unsaturated fatty acids (control). When heated at 150 °C or 200 °C, the amounts of COPs generated were higher in cholesterol samples with linoleic acid than in those with oleic acid. The highest level of COPs produced in samples heated at 150 °C for 10 h was 2.60 mg, while samples heated at 200 °C for 1 h produced 7.43 mg of COPs. Al-Saghir et al. [29] assessed the lipid quality and degree of cholesterol oxidation under different cooking conditions, PUFA was oxidized when heated at high temperatures and consequently produced radicals that oxidize cholesterol. Osada et al. [26] reported that autoxidation of cholesterol may occur in shorter time periods when heated above 120 °C. This indicates that COP production is accelerated during heating at relatively high temperatures in shorter time periods. In addition, Soto-Rodriguez et al. [30] observed levels of cholesterol oxidation products ranging from 4.5 to 5.7 mg/100 g in Mexican fried products. These results suggest that the degree of unsaturation of fatty acids affects cholesterol oxidation. PUFAs, especially *n*-3 PUFA, are attractive compounds to incorporate into functional food, but they are difficult to protect from lipid peroxidation processes. Saldanha and Bragagnolo [31] stated that the amount of PUFA and the grilling temperature both increase the production of cholesterol oxides. Further, the extent of oxidation depends on the composition of fatty acids and minor compounds [32]. We concluded from this study that the temperature affects the rate of cholesterol oxidation and that higher temperatures increase the rates of COP production. Ansorena et al. [33] observed that at 180 °C, the oxidation of unsaturated triglycerides occurs prior to the oxidation of cholesterol and saturated fatty acids. However, the authors reported a contradictory conclusion in which the presence of a lipid matrix increasingly inhibits cholesterol oxidation as the degree of saturation increases.

### COP formation in model systems

**Fig. 1:**
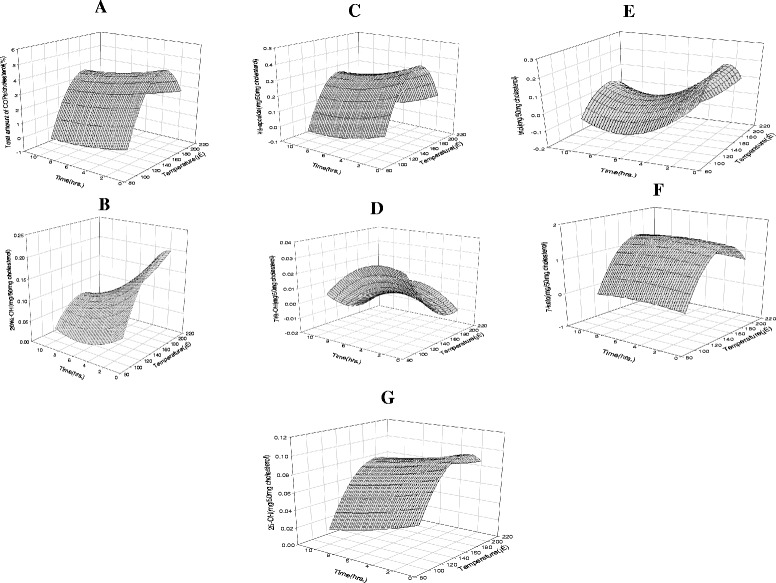
Response surface plots of the content of total COPs (**a**), 20α-OH (**b**), α-epoxide (**c**), 7β-OH (**d**), triol (**e**), 7-keto (**f**), and 25-OH (**g**) in cholesterol heated under various conditions

The linear, quadratic, and interaction coefficients for COP formation in model systems were calculated (data not shown). The coefficients of determination (R^2^) varied from 0.31 to 0.93 for the total amounts of COPs (0.93), 7-keto (0.91), α-epoxide (0.88), 25-OH (0.58), 20α-OH (0.49), triol (0.41), and 7β-OH (0.31). From the R^2^ value of all the COPs, it is evident that strong correlations exist between the heating conditions and COP production, particularly for 7-keto and a-epoxide. Figure 1a shows the response surface 3D mesh plot which illustrates the total amounts of COPs generated under different heating conditions. The production rates of the COPs increased as the heating temperatures were increased, particularly when samples were heated at relatively high temperatures for short time periods. These findings agree with those reported by Osada et al. [26]: heating samples above 120 °C resulted in cholesterol autoxidation in shorter time periods. Cholesterol was converted to COPs during heating and residual cholesterol decreased as cholesterol oxidation increased. These findings of this study are consistent with the results reported by Chien et al. [24]: the authors stated that the percentage of cholesterol changed when samples were heated at 140 °C for up to 2 h. Further, 16.2 % of cholesterol was lost after 40 min of heating and a loss of 86.3 % occurred after heating for 2 h. Zhang [34] also reported a loss of less than 30 % and 70 % of cholesterol after 30 min of heating at 125 °C and 150 °C, respectively. Zardetto et al. [35] studied the formation of COPs in the thermal processing of fresh egg pasta and observed that the total level of COPs increased with both increasing heat treatment and heating time.

The response surface 3D mesh plots corresponding to the amounts of 20α-OH, α-epoxide, 7β-OH, triol, 7-keto, and 25-OH are shown in Fig 1(b-g). High heating temperatures and short heating times resulted in high amounts of 20α-OH in cholesterol (Fig. 1b) and α-epoxide (Fig. 1c). Similar results were reported by Chien et al. [24] that cholesterol oxidation in the solvent system was too fast to be monitored at 160 °C and at higher temperatures. Similarly, α-epoxide and β-epoxide cholesterol may undergo alcoholysis in aqueous media to form cholestane-triol. Chien et al. [25] also observed epoxide formation during an initial period of heating at 150 °C. The amount of 7β-OH generated was high at a low heating temperature and moderate heating time (Fig. 1d). Additionally, the highest level of triol was produced from short heating times (below 2 h) at moderate heating temperatures ranging from 150–170 °C (Fig. 1e). Further, 7-keto (Fig. 1f) was mainly produced at high temperatures (180–200 °C) throughout all heating times (1, 3, 5 and 10 h). In the case of 25-OH, its production gradually increased as the heating time increased (Fig. 1g). These results support findings from previously reported studies [25] in which the percentage of 7-OH (7α-OH and 7β-OH) also increased sharply during the first 10 min of heating at 150 °C and reached a plateau thereafter. Further, a report by Zhang [34] illustrated the changing levels of 7-ketocholesterol when cholesterol was heated at different temperatures and times. For example, 7-ketocholesterol was not detected even after cholesterol had been heated at 125 °C for 30 min. However, 7-ketocholesterol was detected when the heating temperature was raised to 150 °C. The concentration of 7-ketocholesterol drastically increased to 2.11 μg/mL during 30 min of heating at 150 °C. When heated at 175 °C, the concentration rapidly increased to 2.06 μg/mL within 10 min and then leveled off. After 10 min of heating at 200 °C, the 7-ketocholesterol concentration increased continuously and production of 7-ketocholesterol did not correspond to a loss of cholesterol at 200 °C. The rate of cholesterol degradation at 200 °C was lower than the rate of 7-ketocholesterol production after 10 min of heating. Kamal-Eldin [32] reported the effects of temperature on the oxidation of flaxseed oil and observed the following trends: long induction periods at low temperatures (<84 °C), shorter induction periods at intermediate temperature ranges (84-130 °C), and no induction periods at high temperatures (>130 °C). In the current study, it is evident from the 3D surface response plots that COP production increases with increasing temperature, except in the cases of 7β-OH and triol, which increase in production at moderate temperatures; higher temperatures decrease the production levels of 7β-OH and triol.

## Conclusions

Cholesterol oxidation was studied at different heating temperatures and heating times in model systems with controlled pH levels and added unsaturated fatty acids. The results revealed increased cholesterol oxidation at low pH levels and in the presence of either linoleic or oleic acid. The oxidation of unsaturated fatty acid was highest from 1 h of heating, whereas longer heating times resulted in a decrease in cholesterol oxidation. Moreover, the rates of COP production increased as the heating temperatures increased, particularly during shorter heating times. Finally, a heating temperature of 150 °C resulted in maximum cholesterol oxidation and COP production in the model systems.

## Abbreviations

COPs: Cholesterol oxidation products
UFA: Unsaturated fatty acid
PUFA: Polyunsaturated fatty acid
BHT: Butylated hydroxyl toluene
GC: Gas chromatography
SAS: Statistical Analysis System
7β-OH: 7β-hydroxycholesterol
20α-OH: 7α-hydroxy-cholesterol
25-OH: 25-hydroxycholesterol
Triol: Cholestanetriol
α-epoxide: 5,6α-epoxycholesterol
7-keto: 7-ketocholesterol
